# Optimizing the use of expert panel reference diagnoses in diagnostic studies of multidimensional syndromes

**DOI:** 10.1186/s12883-014-0190-3

**Published:** 2014-10-04

**Authors:** Ron L H Handels, Claire A G Wolfs, Pauline Aalten, Patrick M M Bossuyt, Manuela A Joore, Albert F G Leentjens, Johan L Severens, Frans R J Verhey

**Affiliations:** Alzheimer Centre Limburg, School for Mental Health and Neuroscience (MHeNS), Maastricht University Medical Centre, P.O. Box 5800, Maastricht, AZ 6202 The Netherlands; CAPHRI School for Public Health and Primary Care, Faculty of Health Medicine and Life Sciences, Department of Health Services Research, Maastricht University, P.O. Box 616, Maastricht, MD 6200 The Netherlands; Department of Clinical Epidemiology and Biostatistics, Academic Medical Centre, University of Amsterdam, P.O. Box 22700, Amsterdam, DE 1100 The Netherlands; Department of Clinical Epidemiology and Medical Technology Assessment, Maastricht University Medical Centre, P.O. Box 5800, Maastricht, AZ 6202 The Netherlands; Institute of Health Policy & Management, and iMTA, Erasmus University Rotterdam, P.O. Box 1738, Rotterdam, DR 3000 The Netherlands

**Keywords:** Reference diagnosis, Consensus panel, Delphi, Gold standard, Diagnostic validation, Incorporation bias, Multidimensional syndromes, Alzheimer’s disease

## Abstract

**Background:**

In the absence of a gold standard, a panel of experts can be invited to assign a reference diagnosis for use in research. Available literature offers limited guidance on assembling and working with an expert panel for this purpose. We aimed to develop a protocol for an expert panel consensus diagnosis and evaluated its applicability in a pilot project.

**Methods:**

An adjusted Delphi method was used, which started with the assessment of clinical vignettes by 3 experts individually, followed by a consensus discussion meeting to solve diagnostic discrepancies. A panel facilitator ensured that all experts were able to express their views, and encouraged the use of argumentation to arrive at a specific diagnosis, until consensus was reached by all experts. Eleven vignettes of patients suspected of having a primary neurodegenerative disease were presented to the experts. Clinical information was provided stepwise and included medical history, neurological, physical and cognitive function, brain MRI scan, and follow-up assessments over 2 years. After the consensus discussion meeting, the procedure was evaluated by the experts.

**Results:**

The average degree of consensus for the reference diagnosis increased from 52% after individual assessment of the vignettes to 94% after the consensus discussion meeting. Average confidence in the diagnosis after individual assessment was 85%. This did not increase after the consensus discussion meeting. The process evaluation led to several recommendations for improvement of the protocol.

**Conclusion:**

A protocol for attaining a reference diagnosis based on expert panel consensus was shown feasible in research practice.

**Electronic supplementary material:**

The online version of this article (doi:10.1186/s12883-014-0190-3) contains supplementary material, which is available to authorized users.

## Background

Evidence on diagnostic accuracy is often produced in cross-sectional studies by comparing the result of a test under evaluation (the index test, e.g. a newly developed blood test) with the actual presence or absence of a target condition [1]. Ideally, a gold standard to determine the presence of this target condition is available, which is an error-free classification in all patients, blinded from the index test result, and performed within a short interval of time [2]. There are, however, many conditions for which such a gold standard does not exist. In that case, an alternative is then to rely on a clinical reference standard: the best available way for arriving at a clinical classification. One option is then to use a panel of experts who, based on the available information, identify those with the target condition among the persons being tested [2,3].

An example for a disease for which no test fulfils the criteria for a gold standard is Alzheimer's disease (AD). It is defined by a gradual onset of symptoms, deterioration of cognition leading to dementia, and no evidence of another medical comorbidity or medication that could affect cognition [4]. An AD diagnosis is traditionally mainly based on clinical judgement. Recently biomarkers have been given a prominent role in new diagnostic research criteria for AD [4-6] and require validation [7]. Several reference standards have been discussed for AD. A post-mortem neuropathological examination has been criticized for imperfect inter-observer reliability and imperfect association with cognitive impairment or dementia [8,9]. Another reference standard is to follow up a patient in the pre-dementia phase until a clinical diagnosis of AD-type dementia can be made. This requires a long follow-up period to ensure that all patients with a neurodegenerative disease at baseline decline to the level of dementia within that period [10,11].

Several studies have reported on the use of an expert panel to assign a final diagnosis [12-21]. Most studies that rely on an expert panel insufficiently described the rationale behind many of the methodological choices: the basic approach, the number and choice of experts that should be invited, the information that must be supplied to enable expert consensus, the specific questions to be asked, and how to arrive at consensus. Leaving the rationale behind many of these elements unknown makes it difficult to reproduce their findings.

We have developed a protocol for a consensus panel reference diagnosis in AD based on clinically relevant decline as judged by a clinician. We evaluated its feasibility in a pilot project. Based on our findings, we provide a number of recommendations for other researchers considering the use of a consensus panel diagnosis.

## Methods

### Study design

We searched the literature using PubMed for the choices to be made with regard to the methodology of a consensus panel diagnosis. Our protocol was then drafted based on the recommendations from the literature, and tested in a pilot study.

A panel was composed consisting of 3 clinical experts (FV, AL and E. Tan MD) with complementary expertise on neurology, geriatrics and psychiatry. Their clinical experience ranged from 1 to more than 10 years.

**Figure 1:**
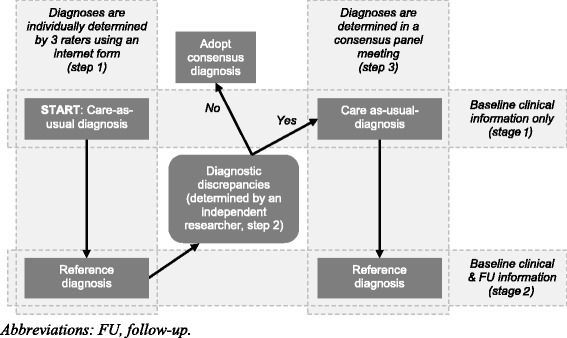
**Process flow of the consensus protocol.** *Abbreviations: FU, follow-up.*

Two diagnoses were set: a care-as-usual diagnosis (reflecting a first visit to a memory clinic) and a reference diagnosis (the best available way to arrive at a clinical classification). For both diagnoses an adjusted Delphi method was applied that started with the assessment of each case by each expert individually, followed by determining diagnostic discrepancies. The discrepancies were then discussed in a consensus meeting between the experts to resolve the discrepancies. This 3-step approach is graphically presented in Figure 1 and explained below.

In the first step, the 3 experts were invited to assess each of the 11 patient cases individually by logging on to a web-based questionnaire. In this questionnaire information on each case was provided in a staged fashion. This step consisted of two *stages*:

1. In *stage* 1, baseline medical history, neurological and physical examination findings, psychiatric and clinimetric assessments, neuropsychological test results, and the results of an MRI scan were summarized in a vignette in tabular format. All three experts were asked to individually answer three questions: 1) “What is the most probable syndrome for this patient?”, 2) “What is the most probable aetiology for this patient?” and 3) “What will be the most likely course of cognitive and/or daily functioning of this patient within 2 years?” (see Table 1). They also indicated their level of diagnostic certainty for each question.

**Table 1:** **Questionnaire used for rating the vignettes**

**Question**	**Response options**	
1a) What is the most probable syndrome for this patient?	Subjective cognitive impairment	
	Mild cognitive impairment	
	Dementia	
1b) How certain are you of this?	Completely uncertain	Completely certain
	0% 10% 20% 30% 40% 50% 60% 70% 80% 90% 100%	
2a) What is the most probable aetiology for this patient?	Alzheimer	
	Vascular	
	Frontotemporal	
	Lewy Bodies	
	Parkinson	
	Other neurodegenerative disease, namely _______________	
	No neurodegenerative disease, namely ________________	
2b) How certain are you of this?	Completely uncertain	Completely certain
	0% 10% 20% 30% 40% 50% 60% 70% 80% 90% 100%	
3a) In your opinion, what will be the most likely course of cognitive and/or daily functioning within 2 years?	Decline	
	Stable	
	Improvement	
3b) How certain are you of this expectation?	Completely uncertain	Completely certain
	0% 10% 20% 30% 40% 50% 60% 70% 80% 90% 100%	

*For the reference diagnosis, question 3a was phrased differently: “In your opinion, what was the course of cognitive and/or daily functioning during the 2-year follow-up?*

2. In *stage* 2, the experts were asked to individually answer the same three questions, though now based on information which included the 2-year follow up of the symptoms that was added to the information from *stage* 1. The same three questions from Table 1 were asked except the last one “What *will be* the most likely course of decline” was rephrased to “what *was* the course of decline”.

See Additional file 1 for an example of the available information to the experts in *stage* 1 and 2. After each stage, the answers were frozen and could not be adjusted retrospectively. No information on biomarkers in cerebrospinal fluid markers, positron emission tomography scans, advanced diffusion tensor, or resting state functional magnetic resonance imaging was provided as this could result in context bias [22].

In the second step, two independent researchers (RH and CW) reviewed the responses. All cases for which all three experts had given identical answers regarding the syndrome, aetiology and prognosis for *stage* 1 as well as *stage* 2 were identified. Levels of certainty for these cases were averaged. All cases for which there was no agreement on any of the three questions were forwarded to the next step.

In the third step, all three experts participated in a face-to-face panel discussion meeting. For each case, a summary of the individual answers to the three questions was presented as well as all relevant clinical information, identical to the one in the first step. The experts were invited to express and exchange their arguments for the answers to the questions and asked to consider whether, in the light of their colleagues’ assessments, they would like to alter their conclusion. A panel facilitator ensured that all participants were able to express their views and encouraged the use of argumentation to arrive at a specific diagnosis, until consensus among all experts was reached. No time limit was set for the discussion.

The care-as-usual diagnosis was defined as the diagnosis based on the information of *stage* 1 at the moment consensus was reached (consensus could have been reached in step 1 before the discussion meeting, because the 3 experts scored identical on the web-based questionnaire, or after the panel discussion meeting in step 3, because the experts scored different in the web-based questionnaire of step 1 and required the discussion meeting to reach consensus). The information at *stage* 1 represented the information available from a first visit to a memory clinic: baseline medical history, neurological and physical examination findings, psychiatric and clinimetric assessments (see Additional file 1).

The reference diagnosis was defined as the diagnosis based on the *stage* 2 information at the moment consensus was reached (consensus could have been reached before the discussion meeting in step 1 or after the panel discussion meeting in step 3). The information at *stage* 2 contained all available information from *stage* 1 at baseline and the 2-year course of symptoms (see Additional file 1).

Afterwards, the experts were asked to complete a process evaluation questionnaire (see Additional file 2) in which they were asked about their experiences regarding the assessment of cases and the consensus discussion, and were asked to provide feedback and suggestions to improve the protocol.

### Patient population

The cases for the evaluation consisted of a sample of 11 patients who had visited the memory clinic of the Maastricht University Medical Centre in the Netherlands in 2009 and 2010 and were suspected of having a primary neurodegenerative disease according to the following eligibility criteria [7]: Mini-Mental State Examination (MMSE) [23] score 20 or higher, Clinical Dementia Rating (CDR) [24,25] between 0 and 1, and availability of a reliable informer or proxy. Subjects were excluded if they had normal pressure hydrocephalus, Huntington’s disease, transient ischaemic attacks or cerebral vascular accidents less than 2 years ago, or a previous psychiatric history. Informed consent was obtained from both the patient and the informal caregiver. Subjects without any follow-up assessment (due to refusal or other reasons) were excluded from this research. The sample was selected such that it included similar proportions of patients with subjective memory complaints, mild cognitive impairment (MCI) and dementia. The hospital’s medical ethics committee approved this study.

### Clinical information

The clinical information included in the vignette (see Additional file 1 for an example) was based on guidelines from the American Academy of Neurology [26] and European Federation of the Neurological Societies [27].

Patient and informant history (medical history, family history, education, co-morbidities, behavioural and psychological symptoms, and activities of daily living) were retrieved from an open interview with both patient and informal caregiver. A neurological and physical examination, and assessment of co-morbidities was performed by a clinician. Clinical tests included the MMSE, CDR, Geriatric Depression Scale-15 (GDS-15) [28], Neuropsychiatric Inventory [29] and Disability Assessment for Dementia (DAD) [30]. Atrophy measurements and white matter lesions were assessed on 3 T MRI scan images by a neuroradiologist. Medial temporal lobe atrophy (MTA) scores, as well as Fazekas scores were used to quantify hippocampal atrophy and the severity of white matter lesions. Neuropsychological examination consisted of a battery of cognitive tests administered by a neuropsychologist. Tests included Rey’s Verbal Learning Test [31,32], Visual Association Test [33], and Digit-Span [34] to assess memory; Letter Digit Substitution Test [35] to assess mental processing rate; and Stroop Color-Word Test [36] and Trail Making Test [37,38] to assess attention, concentration and interference. Raw scores were converted to z-scores, adjusted for age, education level and gender. All assessments took place at baseline and at 12 and 24 months follow-up at the memory clinic, except the MRI scan which was performed only at baseline.

### Statistical analyses

In the analysis we described the care-as-usual and reference diagnoses that were set, and compared the degree of consensus before and after the discussion meeting for the 11 cases. We additionally calculated the average level of confidence in the diagnostic conclusions.

## Results

The baseline patient sample included 8 males and 3 females, with a median age of 78 years (range: 49–86). The median MMSE score was 28 (range: 22–30), CDR was 0.5 (range: 0–1) and DAD was 93% (range: 77–100).

The reference syndrome diagnosis was dementia in 5 cases, MCI in 3 cases, and subjective complaints in 3 cases. There was a 100% consensus on the syndromal diagnosis (see Table 2). Consensus on the reference aetiology diagnoses was reached in 10 cases (91%). These included 8 AD cases and 2 patients without neurodegenerative disease. On 1 case no consensus could be reached (2 experts indicated no neurodegenerative disease while 1 expert indicated a vascular aetiology). The reference statement regarding the course of cognitive and general functioning over time was classified as “improved” in 1 case, “stable” in 2 cases and “declined” in 7 cases by all three experts; in 1 case no consensus could be reached.

**Table 2:** **Confidence and percentage agreement among experts during individual assessment and consensus discussion of 11 cases**

**Item**	**Care-as-usual**		**Reference standard**	
	**Before consensus panel meeting (internet form)**	**After consensus panel meeting**	**Before consensus panel meeting (internet form)**	**After consensus panel meeting**
Degree of consensus (average)	70%	91%	52%	94%
Consensus on syndrome	55%	100%	55%	100%
Consensus on aetiology	82%	100%	64%	91%*
Consensus on disease course	73%	73%	36%	91%
Confidence in the diagnoses (average)	76%	76%	85%	85%

*Mixed Alzheimer and vascular aetiology was scored as either of the two, to facilitate consensus with other experts.

The degree of consensus over all three questions (syndrome, aetiology and disease course) was higher after the panel consensus meeting (91% for the care-as-usual and 94% for the reference standard) compared to before the meeting (70% and 52% respectively). The average level of confidence in the individually established diagnoses was 76% for the care-as-usual diagnosis and 85% for the reference diagnosis. These did not change after the panel discussion meeting.

It took the experts individually on average 6 minutes and 6 seconds to assess a case via the internet form, and 8 minutes and 38 seconds to discuss a discrepant case during the consensus panel meeting.

Table 3 presents the results of the process evaluation questionnaire. Instructions, procedure and diagnostic questions were felt to be clear, except for the difference between the diagnostic question about the *expected* 2-year decline that was asked for the care-as-usual diagnosis and the question about the *actual* observed 2-year decline that was asked for the reference diagnosis (indicated by 1 expert). Insufficient clinical information was reported to be available for several reasons: one expert would have preferred information on clinical history at follow-up, and two experts stated that they would have liked to have a ‘real’ clinical picture or to see the patient in real life. The experts indicated that their reference diagnosis was partly influenced by the concluded baseline care-as-usual diagnosis (diagnostic review bias). Although none of the panel members felt impeded in expressing their opinion during the panel discussion meeting, one expert thought that members had an unequal share in the discussion. The experts also stated that a 2 year follow-up period is sometimes insufficient; it is ‘a compromise between desirable and feasible’ and in an ideal situation they would prefer a longer period.

**Table 3:** **Results of the evaluation questionnaire**

**Item**	**Result**
Instructions, procedure and diagnostic questions were clear	92%
Estimated time per case to fill in the internet form	10 minutes
Diagnosis reflects medical practice	100%
Consensus procedure considered valid to determine a reference diagnosis (scale 0–10)	7.1
Sufficient information available to determine a diagnosis	33%
Influence of baseline diagnosis on reference diagnosis (scale 0–10)	5.7
All panel members had equal shares in the discussion*	50%
Felt impeded in expressing their opinion	0%
Years of experience needed to participate in an expert panel (average, range)*	3, 1-5
Two-year follow-up on disease course is sufficient to determine a reference diagnosis	33%
Three experts is enough	100%

*One expert answered ‘don’t know’ to the this question.

During the panel discussion meeting, the experts indicated that they considered the patient’s history information to be decisive if it contradicted test outcomes of clinical scales such as MMSE or DAD. They also discussed what extent of decline would be sufficient to mark a case as ‘*actual* decline’. They indicated that a patient did not necessarily have to decline to a more severe syndrome, but could also decline to a clinically relevant lower state of cognition and/or functioning within a syndrome label.

## Discussion

In a pilot study, we tested a 3-step protocol for establishing a consensus panel diagnosis using clinical vignettes based on 11 cases in the field of cognitive disorders. The use of an expert panel to attain consensus on a reference diagnosis was considered feasible in research practice.

Two possible forms of bias can occur in a cohort-based diagnostic research as applied in this pilot study: incorporation bias and review bias.

Incorporation bias occurs if information that is used to establish the care-as-usual or biomarker-driven diagnosis is also *used to establish* the reference standard [39]. If for example a patient’s biomarker profile is available to a panel of experts who assign the reference diagnosis it could lead to overestimation of the biomarker’s accuracy, because the association between biomarker and reference standard is artificially inflated. This could easily occur when the standard medical practice diagnosis of AD-type dementia at follow up is used as a longitudinal reference standard, because biomarkers are often used in medical research practice. Many reports on studies that apply such longitudinal medical practice diagnosis as a reference standard provide insufficient information on whether the clinicians were blind for the biomarker results when they set the medical practice diagnosis after having followed up a patient [11]. Incorporation bias can also occur when diagnostic information from care-as-usual is available to the experts when they assign the reference diagnosis. In contrast to the inclusion of a patient’s biomarker profile, as explained above, the care-as-usual baseline information can hardly be omitted since a starting point is required for the reference diagnosis (i.e. the level of decline). It is considered likely that the care-as-usual information provides a ‘small piece of all available information including all follow-ups’ and that the biomarker profile information will receive ‘much weight in the consensus judgement’ when experts set the reference diagnosis [40]. We therefore recommend to incorporate the baseline clinical information but no biomarker information in a reference standard.

**Figure 2:**
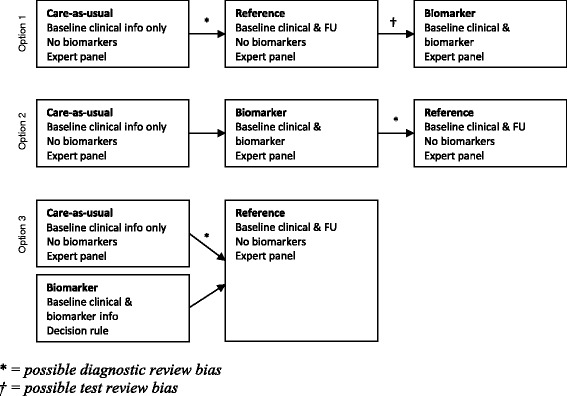
**Single panel approach (option 1 and 2) and partly independent approaches (option 3) to evaluate diagnostic tests for AD.** * = possible diagnostic review bias. † = possible test review bias.

Review bias may occur in two forms. Test review bias occurs when the reference diagnosis is *known* while either the biomarker-driven or care-as-usual diagnosis is being set. Vice versa, when either the biomarker-driven diagnosis or care-as-usual diagnosis is *known* while the reference diagnosis is being set it is called diagnostic review bias [39]. Options 1 and 2 from Figure 2 graphically represent both situations. In an ideal situation, all three diagnoses are assessed by three independent expert panels. Due to limited time and resources a decision rule could be applied that combines the care-as-usual diagnosis with the patient’s biomarker profile using pre-defined cut-off values in a decision rule (see Figure 2 option 3 for an overview) [2]. From our pilot evaluation questionnaire (question 16 of Additional file 2) the experts rated potential review bias an average of 5.7 on a scale of 0 to 10.

Follow-up of pre-dementia patients until a diagnosis of AD-type dementia is a widely applied reference standard in validating biomarkers [11]. When using this reference standard a normal biomarker profile is considered incorrect if a pre-dementia patient does not develop AD-type dementia over time. This approach can lead to an incorrect classification if this patient declines but not sufficiently to reach the state of dementia (i.e. the condition is present but not picked up by the reference standard). On the opposite, an abnormal biomarker profile is considered correct if a pre-dementia patient develops AD-type dementia after a long period of time, for example in 10 years. This approach can be debated in case AD pathology was absent when the subject was tested with the biomarker.

To reduce the extent to which these errors in the reference standard might occur we considered the concept of decline itself, instead of decline to the absolute level of dementia. We do not know what the optimal follow-up time is to establish decline as a valid reference diagnosis. In our opinion, the optimal follow-up period is between 3 and 5 years, as the experts indicated that 2 years may not be sufficient, especially when treatment of cognitive symptoms, e.g. with cholinesterase inhibitors, is started. A follow-up longer than 5 years was considered clinically irrelevant and increase the potential of errors in the reference standard.

The use of a heterogeneous panel composed of experts with different backgrounds, though within the area of interest, has been recommended in the literature [41,42]. Gabel et.al [21] emphasized the importance of selecting experts who are likely to make different types of errors of judgment. Heterogeneity of backgrounds could also help prevent domination by a particular expertise. Other studies applying a consensus diagnosis have used a variety of expertise for their expert panel [17,18]. Gabel et al. [21] found no differences in diagnostic accuracy between a trainee panel and an expert panel though they did not recommend a specific ‘amount’ of required experience. In our study the experts indicated that between 1 and 5 years of experience in the particular field of expertise was necessary to establish a valid diagnosis.

The limited clinical experience of one of our experts might be the reason why one of the other panel members indicated that not everyone had an equal share in the discussion. Perhaps requiring a larger number of years of clinical experience might have helped prevent some of the panel members dominating the discussion. Previous studies showed a large variety regarding the number of experts in the panel, though no specific number was recommended [41,43]. In our study all experts shared the opinion that having 3 panel members was sufficient to establish a valid diagnosis. The qualities of the experts were generally considered more important than the size of the expert panel group. An uneven number of experts could facilitate the decision process if a majority vote is used [3]. Studies reported on in the literature used 2 to 6 experts to determine a consensus diagnosis [13-18,21].

The original Delphi methodology was adjusted in our project since we did not blind the experts from each other’s opinion in the panel discussion [15,42]. Since this might cause bias towards dominant experts, a panel facilitator ensured that all participants were enabled to express their views [21,42]. An alternative, less time–consuming, method could be to adopt a majority decision after individual assessment [13,16,17], which has been reported to have similar diagnostic accuracy as forced consensus [21]. Most studies suggested that experts should apply medical practice diagnostic guidelines, though without imposing strict decision rules.

Video recordings, which could reveal valuable subtle information on patient history [3], were not provided in our study, due to limited time and resources. Instead, we provided a written summary of the clinical history in which an independent researcher had highlighted the most important aspects.

### Recommendations for adjustments to the protocol

Based on the results of the evaluation questionnaire, a number of adjustments to the consensus protocol could be recommended.

- Information on clinical history at follow-up should be included to arrive at a reference diagnosis.
- A 2-year follow-up period for a reference diagnosis that should reflect the best available way for arriving at a clinical classification was considered too short, though the experts could not provide a specific period required.
- Experts invited to the panel should have a minimum of 3 years of clinical experience.
- In some cases the expert panel concluded after the group discussion that no consensus could be reached. As proposed in the literature [13,17], a majority decision could be adopted in these cases to prevent inefficient use of discussion time.
- One expert recognized 1 case from the clinic. It is recommended inviting only experts who have had no direct interaction with the patients under evaluation.
- Initially we included several non-neurodegenerative diagnostic options, which resulted in irrelevant discussions. These were therefore replaced by the question: ‘No neurodegenerative disease, namely…’.
- The login procedure consisted of several steps. When implementing this procedure in research practice, the required time and the complexity of the procedure should be minimized to maximize the willingness of experts to devote their time.

The final protocol can be found in Additional file 3.

### Limitations

Several limitations apply to this study. Not imposing strict decision rules allowed for different views within the panel on how to determine a diagnosis. For example, different sources of information were used to determine an objective memory deficit to distinguish between subjective memory complaints and MCI, and some experts always expected a decline if a neurodegenerative disease was identified. A preparatory discussion among all experts might have reduced discussion time and could increase our understanding of the concept being assessed by the experts.

Another limitation is that the 3-day period that elapsed between filling in the internet form and the consensus panel meeting may have been too short. The experts may have remembered the follow-up information from the internet-based questionnaire when discussing the care-as-usual diagnosis during the consensus panel meeting, which may have resulted in test review bias.

Although the protocol we developed was a practical and transparent method to assign a reference diagnosis, it must be kept in mind that it represents a compromise between available time and resources versus minimisation of bias. The optimal design to evaluate a diagnostic test would be a randomised controlled trial to determine the effects on patient outcome from undergoing the test and the actions taken upon the result. Although no disease-modifying therapies in the pre-dementia phase are available for AD, there is still an interest in the validity of new biomarkers to distinguish disease from non-disease or to enable future planning for patients. When such treatments become available, evidence on the level of diagnostic accuracy can strengthen clinical decision-making [44,45].

## Conclusion

Our aim was to assess the feasibility of establishing a consensus panel diagnosis, for the purpose of studies into cognitive decline and AD, and to establish a protocol for such a consensus panel diagnosis. The protocol was evaluated in a pilot study and the results indicate that this protocol was feasible in research practice.

## Additional files

Additional file 1
**Example fictive patient vignette.**
Additional file 2
**Evaluation questionnaire.**
Additional file 3
**Final proposed protocol.**
