# Supplementary material for: Optimizing the use of expert panel reference diagnoses in diagnostic studies of multidimensional syndromes
Source: BMC Neurol. 2014 Oct 4;14:190. doi: 10.1186/s12883-014-0190-3 (PMC4195860; doi:10.1186/s12883-014-0190-3)
Supplement: Additional file 1 — Example fictive patient vignette. [file 12883_2014_190_MOESM1_ESM.pdf]

Additional file 1: Example fictive patient vignette

STAGE B INFORMATION (available information to set the reference diagnosis)

	STAGE A INFORMATION (available information to set the care-as-usual diagnosis) *Baseline*	*1-year follow-up*	*2-year follow-up*
Age	78		
Gender	female		
Education	Higher Professional Education		
Marital state	Married		
Patient and informal caregiver history	Patient indicates that everything requires a bit more time before things get through to her. Exercise is difficult since her stiff muscles. She indicates her memory is good and concentration during reading is good if there is sufficient interest in the topic. She noticed to loose stuff more often. According to her husband memory and orientation in time has declined. When providing clues she remembers things.	Patient indicates to have trouble hearing. She does not indicate a memory decline. Husband reports further decline of memory, especially short-term memory. Furthermore he things she is disorientated and she does not ask for help by asking things again if she did not hear it well enough.	Patient indicates that memory has declined. Sleeping is ok but she lost appetite. She can't name her daily activities. According to husband and son physical functioning has declined. Orientation is difficult. Husband has to help the patient with many things.
Beginning	Gradual		
Course	Progressive	Progressive	Progressive
1st or 2nd degree relatives with dementia	0		
1st or 2nd degree relatives with Parkinson	1		
1st or 2nd degree relatives with motor neuron disease	0		
Mini-mental State Examination (MMSE)	30/30	26/30	22/30
Clinical Dementia Rating scale (CDR)	0.5	0.5	1
Neuropsychiatric inventory (NPI)	0/144	0/144	0/144
Geriatric depression scale (GDS-15)	1/15	0/15	1/15
Disability assessment for Dementia (DAD)	100% initiative<br>100% planning & organisation<br>94% effective implementation	98% initiative<br>100% planning & organisation<br>91% effective implementation	84% initiative<br>92% planning & organisation<br>76% effective implementation
Disability assessment for Dementia (total score)	98%	96%	84%
Obese	No	No	No
Hypertension	No	No	No
Extrapyramidal Symptoms	No	No	Yes, tremble of hand
Walking difficulties	Yes, small steps	No, walks with small steps	No, walks with small steps
Focal neurological problems	No	No	No
non-fluent speech	No	No	No
other neurological problems	No	No	No
Somatic	Coronary bypass, diabetes	Coronary bypass, diabetes	Coronary bypass, diabetes, hearing problems
Smoker	Never smoked	Never smoked	Never smoked
Amount of tabacco	[not applicable]	[not applicable]	[not applicable]
Drinks alcohol	Yes	[unknown]	Yes
Amount of alcohol per week	5	[unknown]	1
Medication use	Acetylcardio, ascal, metformine	Acetylcardio, ascal, metformine	Acetylcardio, ascal, metformine

	RAW SCORE	Z-SCORE	INTERPRET	RAW SCORE	Z-SCORE	INTERPRET	RAW SCORE	Z-SCORE	INTERPRET
15-Word Learning Task (WLT) total	1+3+4+ 5+6=25	-2.5	Very low	3+4+6+ 6+7=26	-1.6	Low	3+4+4+ 5+3=19	-2.4	Very low
15-WLT delayed recall	4	-1.6	Low	6	-0.60	Below average	3	-1.9	Low
15-WLT retention (trial 5)	4 / 5	0.36	Average	6 / 7	0.68	Above average	0 / 3	-3.7	Very low
15-WLT retention (max. score)	4 / 6	-0.17	Average	6 / 7	0.88	Above average	0 / 6	-3.7	Very low
15-WLT recognition	13-2=11	-0.13	Average	14-1=13	0.23	Average	13-1=12	0.25	Average
Visual Association Test (correct)	12		Average	13		Average	11		Below average
Fluency	25	0.05	Average	11	-2	Very low	12	-1.7	Low
Digitspan	8 / 4		Average	8 / 3		Average	7 / 4		Below average
Visual Object and Space Perception Battery	18 / 9			19 / 10			20 / 10		
Letter Digit Substitution Test (60 seconds)	19	-1.5	Low	20	-1.4	Below average	8	-3.2	Very low
Stroop Color-Word Test 1	57	-1.6	Low	57	-1.6	Low	68.1	-3.2	Very low
Stroop Color-Word Test 2	87	-2.5	Very low	87	-2.4	Very low	103.5	-3.7	Very low
Stroop Color-Word Test 3	180	-2.4	Very low	240	-4.9	Very low	244.2	-4.9	Very low
Stroop interference (3-((2+1)/2))	113	-2.1	Very low	169	-4.6	Very low	195	-4.7	Very low
Trail Making Test A	90	-2.2	Very low	107	-2.5	Very low	108	-2.5	Very low
Trail Making Test B	141	-0.64	Below average	215	-1.7	Low	[unknown]	[unknown]	[unknown]
Trail Making Test B/A index	1.52			2.01			[unknown]		
Conclusion neuropsychological examination	The neuropsychological examination indicates a low mental speed. Memory performance is average except active information uptake.			The patient scores mainly low on tests of mental speed, attention/concentration and executive functioning. Compared to last year several domains declined.			Scores on mental speed are very low though stable compared to last year. Memory declined except recognition.		
Medial temporal lobe atrophy -score left (0-4)	2								
Medial temporal lobe atrophy -score right (0-4)	2								
Global cortical atrophy (0-3)	1								
White matter lesions (Fazekas) (0-3)	1								
Infarcts	0								
Lacunes	0								
Microbleeds	0								
Other clinically relevant findings	[not applicable]								
