# Supplementary material for: Optimizing the use of expert panel reference diagnoses in diagnostic studies of multidimensional syndromes
Source: BMC Neurol. 2014 Oct 4;14:190. doi: 10.1186/s12883-014-0190-3 (PMC4195860; doi:10.1186/s12883-014-0190-3)
Supplement: Additional file 2 — Evaluation questionnaire. [file 12883_2014_190_MOESM2_ESM.pdf]

Additional file 2: Evaluation questionnaire

1. Were the instructions for the internet form clear? *Yes / no, because… / don't know*
2. Was your intended role in the expert panel clear? *Yes / no, because… / don't know*
3. Was the overall procedure clear (combination of internet form and panel discussion)? *Yes / no, because… / don't know*
4. Was the final reference diagnosis a reflection of medical practice? *Yes / no, because… / don't know*
5. Please indicate how much time you spent filling in the internet form: ___ *minutes / don't know*
6. Was the login procedure time-consuming or difficult? *Yes / no, because… / don't know*
7. Was the consensus procedure valid to establish the syndrome? *10-point scale: Completely invalid – completely valid*
8. Was the consensus procedure valid to establish the aetiology? *10-point scale: Completely invalid – completely valid*
9. Was the consensus procedure valid to establish the prognosis? *10-point scale: Completely invalid – completely valid*
10. Were all diagnostic questions clear? *Yes / no, because… / don't know*
11. Was sufficient clinical information available to establish the diagnosis? *Yes / no, because… / don't know*
12. Was all information transparently presented (both in the internet form and the forms used during the panel discussion)? *Yes / no, because… / don't know*
13. Was the sequence of information logical? *Yes / no, because… / don't know*
14. To what extent did the preselection of only the most important information from the patient's history and the neuropsychological conclusion influence your diagnosis? *10-point scale: Not at all – completely*
15. Was it useful to have only the most relevant parts of the history and neuropsychological summary preselected? *Yes / no, because… / don't know*
16. When you were asked to establish the reference diagnosis, your conclusion on the baseline diagnosis was available. To what extent did this influence your reference diagnosis? *10-point scale: Not at all – completely*
17. Did you use the internet during the individual diagnostic scoring? *Yes, namely… / no/ don't know*
18. Did you recognize any of the cases (and thus had foreknowledge about the patient)? *Yes / no / don't know*
19. Each case was assessed by experts from 3 disciplines (neurologist, geriatrician and psychiatrist). In your opinion, did the experts have sufficient expertise to establish a valid diagnosis? *Yes / no, because… / don't know*
20. Did all experts have an equal share in the discussion? *Yes / no, because… / don't know*
21. How many years of experience are required for an expert to establish a valid diagnosis? ___ *years / don't know*
22. Was a follow-up period of 2 years sufficient to establish a valid reference diagnosis? *Yes / no, because… / don't know*
23. Was the number of experts (3) sufficient to establish a valid reference diagnosis? *Yes / no, because… / don't know*

24. Should we have applied stricter decision rules to arrive at a diagnosis (e.g. ask the experts to strictly apply NINCDS-ADRDA or NIA / AA criteria)? *Yes, namely… / no / don't know*
25. Did you feel impeded in sharing your opinion in the group discussion? *Yes, namely… / no / don't know*
26. Do you have any suggestion for improvement of the protocol? *Yes, namely… / no / don't know*
27. Would you be willing to evaluate 50 more cases in a second expert panel? *Yes/no, because…/don't know*
28. Name (optional)
