# Supplementary material for: Optimizing the use of expert panel reference diagnoses in diagnostic studies of multidimensional syndromes
Source: BMC Neurol. 2014 Oct 4;14:190. doi: 10.1186/s12883-014-0190-3 (PMC4195860; doi:10.1186/s12883-014-0190-3)
Supplement: Additional file 3 — Final proposed protocol. [file 12883_2014_190_MOESM3_ESM.pdf]

Additional file 3: Final proposed protocol

- Use a single panel to determine both the care-as-usual diagnosis and the reference diagnosis.
- Do not involve the test(s) under evaluation in this process.
- Compose a panel of three experts with complementary expertise who have had no direct interaction with the patients under evaluation.
- Panellists should have three or more years of medical experience.
- Apply an adjusted Delphi method that starts with the assessment of each case by each expert individually, followed by solving all diagnostic discrepancies during a group discussion meeting.
- The overall process is a 3-step approach:
    - First, experts are invited to assess each case individually by logging on to a web-based questionnaire. All relevant clinical information (in the field of AD-related research: medical history, neurological and physical examination, cognitive assessment, imaging and assessment of co-morbidities at baseline) is presented as concisely as possible in tabular format in a patient vignette. A written summary of the clinical history in which an independent researcher has highlighted the most important aspects is provided. Any diagnostic interpretations (diagnostic conclusions written in the clinical history or neuropsychological examination and biomarker information) are removed from the vignette. Each expert individually answers the three diagnostic questions from table 1 and indicates his/her level of diagnostic certainty. The diagnosis is frozen and cannot be changed during the next steps. Next, the internet form vignette is updated with clinical information from up to 5-year follow-up on all aspects. The same diagnostic questions are asked except the last (what *will be* the most likely course in terms of decline), which becomes 'what *was* the course of decline'.
    - Second, consensus is determined by an independent researcher if all experts separately come to the same conclusions as to the syndrome, aetiology and prognosis, for both the care-as-usual and reference diagnoses. If a discrepancy occurs, all three experts are invited to express their arguments and reach consensus during a discussion meeting. Only the diagnoses in which a discrepancy occurred are carried forward to the next step (e.g. if there is only a discrepancy on the aetiology of the baseline diagnosis, the other diagnostic items syndrome and decline as well as the reference diagnoses are not discussed in the panel meeting).
    - Third, during the group discussion meeting, a summary of the diagnostic conclusions of the three experts is presented to the group (either from the baseline diagnosis or the reference diagnosis). Then, all experts are provided individually with all relevant clinical information (identical to the internet form) and their own diagnostic conclusions. The panel members are asked to consider whether, in the light of their colleagues' assessments, they would like to alter their answers. A panel facilitator ensures that all participants are enabled to express their views and encourages the use of argumentation to arrive at a specific diagnosis, until consensus by all experts is reached. No time limit is set for the discussion. If consensus cannot be reached, the majority diagnosis is adopted.
